# Correction: Efficacy of N-Acetyl Cysteine in Traumatic Brain Injury

**DOI:** 10.1371/journal.pone.0104353

**Published:** 2014-07-28

**Authors:** The PLOS ONE Staff

The first two authors, Katharine Eakin and Renana Baratz-Goldstein, should be noted as contributing equally to this work.
